# Genetic predisposition to celiac disease in Kazakhstan: Potential impact on the clinical practice in Central Asia

**DOI:** 10.1371/journal.pone.0226546

**Published:** 2020-01-02

**Authors:** Dimitri Poddighe, Aida Turganbekova, Dana Baymukasheva, Zhazira Saduakas, Zhuldyz Zhanzakova, Saniya Abdrakhmanova

**Affiliations:** 1 Department of Medicine, Nazarbayev University School of Medicine, Nur-Sultan City, Kazakhstan; 2 RSE on REM «Research and Production Center of Transfusion», Ministry of Health of the Republic of Kazakhstan, Nur-Sultan City, Kazakhstan; King Saud University, SAUDI ARABIA

## Abstract

**Background:**

Celiac disease (CD) is a systemic immune-mediated disorder developing in HLA genetically predisposed individuals carrying HLA-DQ2 and/or HLA-DQ8 molecules. Recent evidences supported a predominant importance of HLA-DQB1 locus and, in particular, HLA-DQB1*02 alleles. This diagnosis is poorly considered in Kazakhstan, because of the assumption that CD is not prevalent in this population.

**Objective:**

To demonstrate that the genetic predisposition to CD in Kazakhstan is not negligible and is actually comparable to Western populations.

**Methods:**

Through the analysis of HLA-DQ genotypes of healthy bone marrow donors from Kazakhstan’s national registry, we estimated the HLA-related genetic predisposition to CD in the country.

**Results:**

We demonstrated that the frequency of CD-related HLA-DQB1 alleles and, as a consequence, of predisposed individuals to CD in Kazakhstan is significant and comparable to countries with the highest disease prevalence.

**Conclusion:**

Considering the dietary style in Kazakhstan, including wheat as a staple food, these results provided a preliminary background of knowledge to expect a significant CD prevalence in Kazakhstan and Central Asia by implementing appropriate and cost-effective diagnostic strategies.

## Introduction

Celiac disease (CD) is a systemic immune-mediated disorder characterized with small intestine atrophic enteropathy, which can manifest with variable clinical patterns of gastrointestinal and extra-gastrointestinal symptoms. [1]

CD is a unique disease in the landscape of autoimmune non-communicable disorders, as regards our knowledge of its etiology: indeed, the necessary HLA genetic background and the necessary environmental trigger to develop the disease at some point in life, have been both well established. In summary, the dietary intake of gluten represents the trigger leading to CD in a small portion of individuals carrying some specific HLA-DQ allelic variants (namely, DQA1*0501-DQB1*02 and DQA1*0301-DQB1*0302), coding class II MHC heterodimers DQ2 and DQ8, respectively. Of course, other concomitant environmental exposures and epigenetic mechanisms are supposed to be critical to determine which individuals will become celiac within a much larger HLA-predisposed population. [2]

Currently, the global prevalence of CD worldwide has been estimated to be around 1% and resulted to be significant and increasing even in areas of the world where CD was previously thought to be negligible [3–5] As regards CD in Asia, several clinical data from Iran, Turkey and Israel suggested a prevalence similar to Europe. Moreover, a significant prevalence of CD has been widely described in the Indian subcontinent, especially in those regions where the dietary exposure to gluten is important. Even in China CD diagnoses have been growing up in recent years, because of the introduction of Western-like dietary styles. Unfortunately, no epidemiological studies are currently available from Central Asia, although wheat consumption is comparable to Western countries. Here, a poor awareness about CD and its clinical expression (which may be subtle and dominated by extra-gastrointestinal manifestations) and the baseless belief that CD is not prevalent in these populations, have hampered the clinical approach and research regarding this specific non-communicable disease so far. [3, 6–7]

Very recently, we reviewed those few scientific sources available from the Central Asia about pediatric CD and, actually, we obtained cues that CD could not be as negligible as thought so far. However, we could not find any specific study providing data on the genetic predisposition to CD development in Central Asian populations. [7] Here, we describe the allelic frequencies at the HLA-DQB1 locus in healthy bone marrow donors from Kazakhstan, in order to first demonstrate that the HLA genetic predisposition in the general population of this geographical area is comparable to that in Europe and North America.

## Materials and methods

In order to implement a National Registry of potential bone marrow donors for hematopoietic stem cell transplants, > 4,500 people with Kazakh citizenship (age range: 18–45 years; approximately 45% female and 55% male) have been HLA genotyped at the Research and Production Center of Transfusion (Nur-Sultan, Kazakhstan) between September 2011 and December 2015. At the time of their voluntary enrollment in this registry, all healthy donors signed an informed consent to undergo HLA analysis and immune phenotyping.

In this study, we analyzed the distribution of the allelic variants of the HLA system, taking advantage of these existing data from the pool of bone marrow donors, which can be considered representative of the general adult population in Kazakhstan for our study objective, aiming to assess the immune-genetic predisposition to CD in this country. Therefore, no additional tests and/or experiments have been carried out for the purposes of the present study, and the research group received only a completely anonymized dataset including the available HLA typing. In detail, a completely anonymized dataset including only class I and class II HLA results was provided to perform our analysis and study. This dataset included the results from 4,580 people, but a complete HLA-DQB1 genotype was available for 3,980 individuals only. Indeed, the study objective was to analyze the population frequency of the HLA-DQB1 allelic variants known to predispose to CD and, then, how many people are carriers of HLA-DQB1 genotypes at risk for CD, in the population of the Republic of Kazakhstan.

This study is part of a general research project analyzing anonymized secondary HLA data in the Kazakh population, which has been officially approved by the Institutional Research Ethics Committee (IREC) of the Nazarbayev University (Nur-Sultan, Kazakhstan) with approval of 25-02-2019. This study has been approved as exempted from IREC oversight, since “this research involves the collection or study of existing data that are recorded by the investigator in such a manner that subjects cannot be identified, directly or through identifiers linked to the subjects”. Although the present study did not include any biological test (as already explained), here are the material and methods by which the HLA-A*, B*, C*, DRB1*, DQB1* loci were previously genotyped. For the determination of the high-resolution HLA genotype at aforementioned loci, the genomic DNA was isolated from blood leukocytes by column method, using the PROTRANS DNA BOX 500 reagent kit (PROTRANS medizinishe diagnostische produkte Gmbt, Mannenheim, Germany). DNA concentration was measured by NanoDrop 2000 spectrophotometer (Thermo Fisher Scientific, Finland), in order to ascertain an amount in the range between 50–100 ng/ml. [8] The high-resolution typing was carried out by using primers (for HLA-A*, B*, C*, DRB1*, DQB1* loci) provided by the company PROTRANS Diagnostics (medizinishe diagnostische produkte Gmbt, Mannheim, Germany). At the stage of DNA amplification by polymerase chain reaction (PCR), the haplotypes were separated, and hemizygous sequencing was performed. Sequencing was performed in both forward and reverse directions on exons 2, 3 and 4 for loci HLA-A*, B* and C*, exon 2 for locus DRB1*, exons 2 and 3 for DQB1* locus. [9] Nucleotide sequencing was performed through capillary electrophoresis by using BigDye v1.1. terminator reagents (Applied Biosystems, Foster City, CA, United States) and by using the 24-capillary "3500XL" and 96-capillary "3730XL” genetic analyzers (Applied Biosystems, Foster City, CA, United States). Sequencing results were analyzed through the Sequence Pilot Software (JSI medical systems, Germany) and the HLA alleles were identified according to the international database IMGT/HLA database. [10]

## Results

This retrospective analysis of existing (secondary) data provided an overview on the HLA-DQB1 polymorphisms in the population of the Republic of Kazakhstan. The main endpoint of this study was to assess the immune-genetic predisposition of this population to develop CD, by exploiting and analyzing the existing HLA-DQB1 dataset. Therefore, this study provides a preliminary background of knowledge to plan and support future investigations about the prevalence of CD in Kazakhstan, especially in children.

Our analysis demonstrated that the frequency of HLA-DQB1 alleles predisposing to CD in Kazakhstan’s healthy blood donors is comparable to Western populations, as showed in Tables 1 and 2.

**Table 1:** HLA-DQB1 genotype of 3,980 bone marrow healthy donors from Kazakhstan (N: absolute number of copies in the population; F: allelic frequency in the population).

HLA-DQB1*	N	F (%)
**02:01**	1,269	15.94
**02:02**	381	4.79
**02:03**	5	0.06
**03:01**	1,705	21.42
**03:02**	648	8.14
**03:03**	411	5.16
**03:04**	24	0.30
**03:05**	18	0.23
**03:12**	2	0.03
**03:13**	1	0.01
**04:01**	107	1.34
**04:02**	206	2.59
**04:03**	1	0.01
**04:04**	1	0.01
**04:19**	1	0.01
**05:01**	79**3**	9.96
**05:02**	321	4.03
**05:03**	221	2.78
**05:04**	30	0.38
**05:05**	3	0.04
**05:09**	1	0.01
**06:01**	346	4.35
**06:02**	723	9.08
**06:03**	482	6.06
**06:04**	149	1.87
**06:05**	11	0.14
**06:07**	1	0.01
**06:09**	93	1.17
**06:11**	2	0.03
**06:14**	3	0.04
**06:15**	1	0.01
**TOTAL**	**7,960**	100

**Table 2:** Distribution of CD-predisposing HLA-DQB1 alleles in the bone marrow healthy donors in Kazakhstan (N: number of carriers; %: carrier percentage of the whole cohort of donors).

Class II MHC β chain	HLA-DQ B1*(1)	HLA-DQ B1*(2)	N	%
	02:01	02:01	86	2.16
	02:01	02:02	43	1.08
	02:02	02:02	21	0.53
**β**_**DQ2**_**+β**_**DQ2**_		**Sub-Total**	**150**	**3.77**
	02:01	03:01	275	6.91
	02:01	03:03	75	1.88
	02:01	03:04	2	0.05
	02:01	03:05	1	0.03
	02:01	04:01	14	0.35
	02:01	04:02	35	0.88
	02:01	04:04	1	0.03
	02:01	04:19	1	0.03
	02:01	05:01	119	2.99
	02:01	05:02	52	1.31
	02:01	05:03	49	1.23
	02:01	05:04	9	0.23
	02:01	05:09	1	0.03
	02:01	06:01	53	1.33
	02:01	06:02	129	3.24
	02:01	06:03	94	2.36
	02:01	06:04	27	0.68
	02:01	06:05	1	0.03
	02:01	06:09	15	0.38
	02:02	03:01	91	2.29
	02:02	03:03	9	0.23
	02:02	04:01	5	0.13
	02:02	04:02	14	0.35
	02:02	05:01	36	0.90
	02:02	05:02	19	0.48
	02:02	05:03	9	0.23
	02:02	06:01	12	0.30
	02:02	06:02	35	0.88
	02:02	06:03	18	0.45
	02:02	06:04	8	0.20
	02:02	06:09	8	0.20
**β**_**DQ2**_		**Sub-Total**	**1217**	**30.58**
	02: 01	03:02	101	2.54
	02:02	03:02	32	0.80
**β**_**DQ2**_**+β**_**DQ8**_		**Sub-Total**	**133**	**3.34**
	03:02	03:02	28	0.70
**β**_**DQ8**_**+β**_**DQ8**_		**Sub-Total**	**28**	**0.70**
	03:02	02:03	2	0.05
	03:02	03:03	37	0.93
	03:02	03:04	3	0.08
	03:02	03:05	4	0.10
	03:02	04:01	10	0.25
	03:02	04:02	18	0.45
	03:02	05:01	83	2.09
	03:02	05:02	20	0.50
	03:02	05:03	14	0.35
	03:02	05:04	3	0.08
	03:02	06:01	23	0.58
	03:02	06:02	59	1.48
	03:02	06:03	37	0.93
	03:02	06:04	16	0.40
	03:02	06:09	9	0.23
**β**_**DQ8**_		**Sub-Total**	**338**	**8.49**
**TOTAL**			**1,866**	**46.88%**

Briefly, the allelic frequency (F) of the HLA-DQB1*02:01 and 02:02 coding the β chain of MHC-DQ2 heterodimer, was 20.73% overall (Table 1). 1,528 people (out of 3,980 donors, 38.39% of the whole cohort) carry at least one copy of HLA-DQB1*02 allele and, among them, 150 donors (3.77%) resulted to be homozygous (Table 2). The exact allelic frequencies of HLA-DQB1*02:01 and HLA-DQB1*02:02 were 15.94% and 4.79%, respectively. (Table 1).

The allelic frequency of HLA-DQB1*03:02, coding the β chain of MHC-DQ8 heterodimer, was 8.14% (Table 1). 499 people (out of 3,980 donors, 12.53% of the whole cohort) carry at least one copy of HLA-DQB1*03:02 allele and, among them, 28 donors (0.7%) resulted to be homozygous (Table 2).

Finally, 133 patients (3.34% of the whole cohort) resulted to be double heterozygous, as they carry one copy of both HLA-DQB1*02 and HLA-DQB1*03:02 (Table 2).

## Discussion

CD is almost completely unexplored in some areas of the world, including large part of Asia. Here, except for Indian subcontinent and Western Asia, there are few available studies and, in particular, this issue has been never specifically addressed in Central Asia, including Kazakhstan. Indeed, other public health issues (related to some communicable diseases) have attracted a lot of resources and attention in Kazakhstan in last few years; moreover, as regards CD specifically, it has been—and it is still—considered a negligible disease in this country and, thus, such a diagnosis is usually sought only in presence of severe gastrointestinal manifestations without any other explanation, according to our personal clinical observation so far. [6–7] However, the dietary style in this country includes wheat as staple food: indeed, the average consumption per person per year of wheat foods in Kazakhstan, has been estimated to be 100–150 kg. [6,11] Therefore, the exposure to this necessary environmental trigger for CD in Central Asia is comparable to Western countries, where the disease prevalence in the general population is known to be around 1% and the portion of HLA-DQ CD-predisposed individuals is comprised between 30–40%. [2]

As previously mentioned, studies investigating the epidemiological burden of CD in Kazakhstan are currently missing, and a number of medical (e.g. poor awareness of the huge clinical heterogeneity of CD), diagnostic (availability of inappropriate and/or poorly accessible serological screening methods) and economical (cost of the diagnostic procedures charged to the patient/family, etc.) barriers have hampered the appropriate clinical approach to this disease in this country. [7]

Therefore, an important preliminary step to improve and support the correct diagnosis and clinical research on CD in Kazakhstan and, in general, in Central Asia, is to demonstrate that CD could be as likely as in Western countries. As discussed above, the dietary style is comparable to Europe and North America in terms of staple foods (including wheat) and, thus, similar strategies of screening and diagnosis may deserve to be implemented, as long as there are data demonstrating that the population of Kazakhstan has a similar HLA background of predisposition to CD as other populations, in which the disease prevalence has been showed to be considerable and the dietary regimen is comparable.

In order to accomplish this goal, we analyzed the secondary data coming from the HLA registry of bone marrow donors in the Republic of Kazakhstan and, importantly, we demonstrated that they have allelic frequencies of HLA-DQ CD-predisposing genes comparable to European populations. In detail, around 38% and 12.5% of healthy blood donors resulted to respectively carry HLA-DQB1*02 and/or HLA-DQB1*03:02 alleles, which encode the β chain of class II MHC heterodimers (respectively, DQ2 and DQ8) known to represent the necessary (but not sufficient) immune-genetic background to develop CD.

Unfortunately, the HLA-DQA1 analysis is not available, since it was not performed for the original purpose of this HLA genotyping, namely the implementation of the bone marrow donor registry. Consequently, we cannot report how many people actually express the full MHC-DQ2/DQ8 heterodimers. Anyway, the available data related to HLA-DQB1 locus can support our original hypothesis, as discussed below.

First, we compared our results (between brackets) with some studies coming from Western countries, such as the United States of America (USA), for instance. Recently, Moore E et al. published the HLA genotype analysis of 496 healthy adult donors from that country. These authors found a value of 19.3% as allelic frequency of HLA-DQB1*02 in their population, which is close to our results (20.7%). Moreover, the number of individuals carrying this allelic variant was 36.8%, which is again comparable to our findings (38.39%). As for HLA-DQB1*03:02, the allelic frequency and the percentage of carriers were 9% (vs. 8.14%) and 17.7% (vs. 12.5%), respectively. [12]

Previously, a larger study by Pietzak MM et al. assessed the HLA-DQ genotype of 10,191 consecutive blood samples analyzed in their HLA laboratory: they found results positive for HLA-DQB1*02 or HLA-DQB1*03:02 in 43.1% (vs. 38.39%) and 14.9% (vs. 12.5%) of cases, respectively. Therefore, they retrieved percentage values of individual carrying at least one copy of the two HLA-DQB1 predisposing alleles that is comparable to our results. [13]

These comparisons showed that the allelic frequencies and the number of carriers of these specific allelic variants predisposing to CD in the population of Kazakhstan, are similar to the USA, where the prevalence of CD is known to be around 1%. [3] Therefore, this population may be considered as having the same HLA-related immune-genetic risk of CD as Western populations.

As mentioned, our analysis is limited by the absence of the concomitant HLA-DQA1 genotype, which would indicate how many people, among those carrying the β chain of HLA-DQ2 and/or -DQ8 molecules, are effectively able to express the full DQ2 and/or DQ8 heterodimers.

However, several clinical studies provided evidences about the predominant role of the β chain in determining the risk for CD. Megiorni et al. depicted a risk gradient by which patients being “HLA-DQ2/DQ8 heterozygous” and “DQ2 homozygous” showed the highest risk and, to follow, patients being “DQ8 homozygous”, “DQ8 heterozygous” or “DQ2 heterozygous” and, then, people carrying a double dose of HLA-DQB1*02 only. Importantly, this last group of patients showed a significant risk to develop CD anyway, compared to the general population, although they expressed neither the complete DQ2 nor -DQ8 heterodimer. [14–15] More recently, our group published two meta-analyses and one observational study, supporting this concept: a double dose of HLA-DQB1*02 resulted to be associated with the highest risk to develop pediatric CD (OR>5), regardless of other HLA-DQ alleles; moreover, even a single copy of HLA-DQB1*02 resulted to confer a significant risk to develop CD (OR≅4). [16–17] The analysis of other published studies providing precise data about the HLA-DQ genetic background in CD patients could further support this aspect. [18–20]

Additionally, to further strengthen our thesis, we retrieved two previous HLA studies from Kazakhstan, which actually included much fewer individuals. Kuranov AB et al. carried out a study (including 233 Kazakh people) to compare class II HLA alleles between patients with drug resistant tuberculosis (TB, n = 76) and healthy controls (n = 157) in Kazakhstan. By analyzing their aggregated data, we calculated that 42.11% of TB patients and 40.76% of their healthy controls were carriers of HLA-DQB1*02; as for HLA-DQB1*03:02, the carrier frequency was 13.16% and 14.01%, respectively. These data are in agreement with our findings, but the main value of this study for our purpose is that these authors provided also the HLA-DQA1 genotyping: HLA-DQA1*05:01 (coding the α chain of DQ2 heterodimer) and HLA-DQA1*03:01 (coding the α chain of DQ8 heterodimer) were 11.59% and 21.46%, respectively. [21] Kuranov AB et al. published another study showing even the allelic frequencies of those HLA-DQ loci, which resulted to be 9.6% and 13.1% for HLA-DQA1*05:01 and -DQA1*03:01, respectively. As for HLA-DQB1 loci, the allelic variants *02 and *03:02 showed respective frequencies of 23.8% and 7.5%, which once again are comparable with our results from a much larger cohort of patients coming from the entire Kazakhstan and not from one city (the capital Nur-Sultan City) only. [22]

## Conclusion

All these findings and discussion support the concept that the HLA-related genetic predisposition to CD in the general population of Kazakhstan is not negligible, and it is actually comparable to countries where CD is quite prevalent. Considering the Western-like dietary style (at least as regards the intake of foods containing wheat), it is reasonable to expect that Kazakhstan will show comparable prevalence rates of CD as Europe and North America. Therefore, appropriate diagnostic and/or screening procedures for CD should be implemented in Kazakhstan and Central Asia, especially for children, who might be more likely to develop persistent CD-related complications, due to the disease onset during their growth and their longer life expectancy.
